# A Perspective on Quality Evaluation for AI-Generated Videos

**DOI:** 10.3390/s25154668

**Published:** 2025-07-28

**Authors:** Zhichao Zhang, Wei Sun, Guangtao Zhai

**Affiliations:** Department of Electronic Engineering, Shanghai Jiao Tong University, Shanghai 200030, China; liquortect@sjtu.edu.cn (Z.Z.);

**Keywords:** video quality assessment, AI-generated video, MLLM

## Abstract

Recent breakthroughs in AI-generated content (AIGC) have transformed video creation, empowering systems to translate text, images, or audio into visually compelling stories. Yet reliable evaluation of these machine-crafted videos remains elusive because quality is governed not only by spatial fidelity within individual frames but also by temporal coherence across frames and precise semantic alignment with the intended message. The foundational role of sensor technologies is critical, as they determine the physical plausibility of AIGC outputs. In this perspective, we argue that multimodal large language models (MLLMs) are poised to become the cornerstone of next-generation video quality assessment (VQA). By jointly encoding cues from multiple modalities such as vision, language, sound, and even depth, the MLLM can leverage its powerful language understanding capabilities to assess the quality of scene composition, motion dynamics, and narrative consistency, overcoming the fragmentation of hand-engineered metrics and the poor generalization ability of CNN-based methods. Furthermore, we provide a comprehensive analysis of current methodologies for assessing AIGC video quality, including the evolution of generation models, dataset design, quality dimensions, and evaluation frameworks. We argue that advances in sensor fusion enable MLLMs to combine low-level physical constraints with high-level semantic interpretations, further enhancing the accuracy of visual quality assessment.

## 1. Introduction

The rapid evolution of AI-generated content (AIGC) has reshaped numerous industries [1]. With the advancement of sensor technology, more and more generative models can create highly realistic and rich videos from multiple modal inputs such as text, images, videos, and even depth information. These breakthroughs are largely driven by diffusion- and transformer-based generative models, whose unprecedented capacity for spatio-temporal synthesis now rivals professional production pipelines. Yet, despite these significant strides, assessing the quality of AIGC videos remains a formidable challenge. Unlike traditional media, where quality can often be gauged with well-established full-reference or no-reference metrics, AIGC videos exhibit failure modes that existing measures were never designed to capture: inconsistent motion, object disocclusion, semantic drift between text prompts and visuals, and subtle perceptual artifacts introduced during iterative denoising. Consequently, developing robust and reliable video quality assessment (VQA) methods for AIGC has become a pressing concern for both researchers and industry practitioners.

Traditional VQA techniques initially relied on full-reference metrics. These metrics, including PSNR, SSIM, and VMAF, quantify pixel-level fidelity by comparing a distorted video to a pristine ground truth. These methods implicitly assume both the availability of a reference signal and distortion patterns that stem from compression or capture noise. Neither assumption is valid for AIGC. Generated videos lack a natural reference, and their artifacts tend to appear as higher-order semantic inconsistencies. These inconsistencies include issues such as object disocclusion, identity swaps, and prompt drift, rather than simple signal degradation. As a result, full-reference indices systematically underestimate or misinterpret the true perceptual impact of generative errors.

To remove the dependency on reference clips, the field then embraced no-reference (blind) CNN-based and ViT-based models. Architectures built on ResNet [2], I3D [3], SlowFast [4], or Vision Transformers [5] learn to regress quality scores directly from visual content, capturing texture, motion blur, and compression artifacts more flexibly than handcrafted indices. Nevertheless, these networks are typically trained on UGC or broadcast distortions and thus struggle with AIGC-specific failure modes: they have limited awareness of text–video alignment, often treat semantic implausibility as acceptable variation, and require large amounts of labeled data that are costly to obtain for every new generative paradigm.

**Figure 1:**
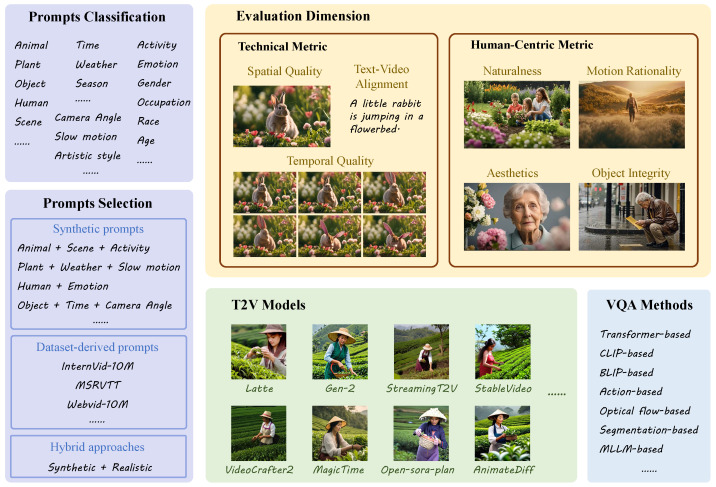
Illustration of the AIGC video quality assessment development era.

The latest stage is marked by multimodal large language models (MLLMs), such as Qwen-VL [6], InternVL [7], and GPT-4V [8], which jointly embed appearance, motion, and language semantics. By conditioning on the original prompt, MLLMs holistically evaluate spatial fidelity, temporal coherence, and textual alignment within a single pre-trained backbone, achieving correlations with human opinion that rival or surpass earlier methods while providing interpretable language rationales. Complementing this shift, unsupervised and semi-supervised strategies—e.g., synthetic perturbations, self-distillation, and lightweight adapters—dramatically reduce the need for exhaustive subjective labels, making MLLM-centric pipelines scalable and adaptable across domains. Figure 1 summarizes the historical evolution of AIGC-VQA.

Against this backdrop, we advance a central perspective: MLLM-based methods will supersede handcrafted metrics and purely CNN and ViT architectures, becoming the de facto standard for AIGC VQA. This claim rests on three observations. (1) MLLMs unify vision, language, and temporal reasoning within a single pre-trained backbone, allowing simultaneous evaluation of spatial fidelity, motion continuity, and semantic alignment. (2) Prompt engineering and lightweight adapters enable rapid specialization to new distortion types or downstream tasks with minimal additional data. (3) Early benchmarks on datasets such as AIGV-Assessor [9], Q-Eval-100K [10], and Q-Bench-Video [11] already show that MLLM predictions correlate with mean opinion scores better than previous state-of-the-art metrics while also providing natural-language rationales that enhance interpretability.

## 2. AIGC Video Generation

With the rapid development of artificial intelligence, video generation—especially text-to-video (T2V) generation—has made significant progress in recent years, as shown in Table 1. Broadly, T2V models can be categorized into distinct frameworks: autoregressive-based [12,13,14,15,16,17] and diffusion-based [18,19,20,21,22,23,24,25,26,27,28] approaches. Each framework embodies a different paradigm for modeling the complex spatial and temporal dynamics inherent in video synthesis and presents its own unique set of advantages and challenges.

Subsequent research shifted towards autoregressive-based models, which leverage the sequential modeling capabilities of transformers. For instance, NÜWA [12] leverages a 3D transformer encoder–decoder with a nearby attention mechanism for high-quality video synthesis. NÜWA-Infinity [13] presents a “render-and-optimize” strategy for infinite visual generation. CogVideo [14] utilizes pre-trained weights from the text-to-image model and employs a multi-frame-rate hierarchical training strategy to enhance text–video alignment. Phenaki [15] uses a variable-length video generation method with a C-ViViT encoder–decoder structure to compress video into discrete tokens. Although autoregressive models benefit from the powerful sequence modeling inherent in transformers, their performance is highly contingent upon the quality of the discrete tokenization process, and they can struggle to maintain fine-grained details if the VQ-VAE is not sufficiently expressive.

More recently, diffusion-based [54] models have emerged as the dominant framework for T2V generation [55,56,57]. These methods [18] extend the principles of denoising diffusion probabilistic models from the image domain to video. Diffusion-based T2V models, such as AnimateDiff [46], VideoCrafter [24], Text2Video-Zero [27], Tune-a-Video [12], and LVDM [19], typically operate by learning a latent space representation through an autoencoder and then iteratively denoising a random noise sample into a coherent video.

The Video Diffusion Model [18] applies the diffusion model to video generation using a 3D U-Net architecture combined with temporal attention. To reduce computational complexity, LVDM [19] introduces a hierarchical latent video diffusion model. Gen-1 [37] is a structure and content-guided video diffusion model, training on monocular depth estimates for control over structure and content. Tune-a-video [20] employs a spatiotemporal attention mechanism to maintain frame consistency. Video Crafter1 [24] uses a video VAE and a video latent diffusion process for lower-dimensional latent representation and video generation. NÜWA-XL [28] uses two diffusion models to generate keyframes and refine adjacent frames. To ensure temporal consistency, these models incorporate additional conditioning mechanisms—such as cross-frame attention or motion guidance—that explicitly account for the relationships between successive frames. While diffusion-based approaches excel in generating high-fidelity frames that closely adhere to the provided textual descriptions, they are often computationally expensive due to the iterative nature of the sampling process and may require sophisticated strategies to maintain temporal coherence over longer sequences.

The evolution of T2V generation techniques reflects a progression from early GAN/VAE-based methods, which laid the initial foundation by jointly modeling static and dynamic components, to autoregressive-based approaches that exploit the sequential modeling power of transformers and finally to diffusion-based models that have recently achieved state-of-the-art frame quality and conditioning flexibility. Each of these frameworks contributes unique insights and capabilities to the field, and ongoing research continues to explore hybrid strategies that might combine their respective strengths to enhance further the fidelity, coherence, and controllability of text-to-video generation.

## 3. Text-to-Video Quality Assessment Benchmarks

### 3.1. Prompts Selection

The design of text prompts plays a pivotal role in evaluating text-to-video (T2V) generation models, as it directly impacts the diversity, controllability, and real-world relevance of generated videos. Existing studies have adopted distinct strategies for prompt construction, broadly categorized into synthetic prompts, dataset-derived prompts, and hybrid approaches; the characteristics of each scheme are shown in Table 2.

A significant body of work leverages manually crafted or MLLM-augmented prompts to systematically test model capabilities. For instance, T2VBench [58] synthesizes prompts hierarchically using Wikipedia concepts and LLMs, covering 16 temporal dynamics such as causal chains and geometric transformations. Similarly, T2V-CompBench [59] designs 700 prompts with combinatorial challenges (e.g., attribute binding, multi-object counting) to stress-test compositional reasoning. GAIA [60] and Human-AGVQA [61] focus on human-centric scenarios, curating prompts for body/hand movements and social interactions to assess action plausibility. These approaches prioritize controlled diversity but may lack alignment with real-world user intents.

Other studies extract prompts from existing text–video datasets to ensure real-world relevance. DEVIL [62] utilizes MSR-VTT and ActivityNet captions, further refining them via GPT-4 and human annotation to categorize prompts by motion intensity (low/medium/high dynamic). AIGV-Assessor [9] combines prompts from MSR-VTT with orthogonal categories (e.g., spatial objects, temporal events) to balance realism and coverage. While such methods benefit from grounded descriptions, they often inherit dataset biases and limited creativity.

Recent efforts integrate synthetic and real-world prompts to bridge the gap between controllability and authenticity. VBench [63] merges human-designed prompts with dataset-derived examples, organizing them into eight thematic categories (e.g., animals, architecture) and 16 quality dimensions (e.g., motion smoothness, temporal consistency). FETV [64] augments open-domain datasets with manually authored prompts emphasizing temporal logic (e.g., “a car accelerates while turning left”), achieving a balance between complexity and practicality. Notably, T2VQA-DB [58] crowdsources prompts from users and augments them with structured dataset entries, enabling large-scale evaluation of real-world applicability.

Certain frameworks target niche dimensions. TC-Bench [65] designs prompts with explicit start–end states (e.g., “daytime to sunset transition”) to quantify temporal coherence, while LGVQ [66] structures prompt around foreground–background interactions to test spatial–temporal disentanglement. EvalCrafter [67] employs LLMs to expand user-provided prompts into fine-grained variations, enhancing coverage of everyday scenarios.

Despite these advances, current methods face challenges in scalability (e.g., small prompt sets in GAIA [60]) and holistic coverage. Most frameworks prioritize either temporal dynamics or compositional accuracy, with few addressing both. Additionally, prompts derived from real-world data often lack granular metadata (e.g., motion intensity labels), limiting their utility for diagnostic evaluation.

### 3.2. Evaluation Dimensions

The general evaluation dimensions focus on ensuring that all videos maintain high levels of overall quality, spatial consistency, temporal consistency, and accurate alignment with textual descriptions. These dimensions are fundamental to any video generation task, ensuring that the videos are coherent, stable, and consistent across frames and time. On the other hand, the special content evaluation dimensions address the specific challenges associated with generating videos that involve human actions, complex interactions, or dynamic scenes. These dimensions evaluate the plausibility of actions, the accuracy of human representation, and the correct depiction of object relationships and behaviors, which are essential for generating content that is both realistic and faithful to the provided text prompt.

#### 3.2.1. General Evaluation Dimensions

This category encompasses evaluation dimensions applicable to all types of videos, regardless of content, with a focus on overall video quality, spatial consistency, temporal consistency, and the alignment between text prompts and generated video content.

Overall quality assesses the visual fidelity and perceptual quality of the generated video. It considers how coherent and natural the video appears to the viewer, focusing on factors such as clarity, absence of visual artifacts, and overall aesthetic appeal. Several datasets such as T2VQA [68], LGVQ [66], AIGV-Assessor [9], and Human-AGVQA [61] have been designed to evaluate this dimension, emphasizing the quality and realism of the generated videos without introducing perceptual distortions.

Spatial quality focuses on the consistency of objects and scenes in the video, ensuring that spatial relationships are accurately maintained across frames. This includes the positioning and movement of objects, particularly in dynamic or interactive scenes, where objects must remain in context and correctly aligned. In FETV [64], LGVQ [66], AIGV-Assessor [9], etc., the evaluation of this dimension is central to datasets that assess how well-generated videos maintain spatial consistency and avoid inconsistencies in the depiction of objects within the video.

Temporal quality evaluates the smoothness and consistency of video transitions over time. It includes assessing the continuity of motion and the smoothness of scene transitions, ensuring that there are no unnatural jumps or flickers. This dimension in T2VBench [58], DEVIL [62], TC-Bench [65], EvalCrafter [67], etc., is crucial for evaluating how well videos maintain temporal consistency, particularly in complex or fast-moving scenes, as demonstrated by datasets that focus on dynamic scene generation.

Text–video alignment measures how accurately the generated video aligns with the textual prompts. This dimension evaluates whether the video content faithfully reflects the details described in the prompt, including the accurate representation of objects, actions, and relationships. In LGVQ [66], AIGV-Assessor [9], and VBench [63], the alignment between text and video is critical for ensuring that the model generates content that matches the description provided, and several datasets specifically assess the quality of this alignment in terms of both semantic and visual accuracy.

#### 3.2.2. Special Content Evaluation Dimensions

This category addresses evaluation dimensions tailored to specific types of videos, particularly those involving more specialized content, such as human activities or complex dynamic actions and interactions.

Action plausibility, particularly in videos involving human figures or animals, evaluates the realism and physical feasibility of actions. This dimension assesses whether the actions performed in the video, such as running or jumping, are physically possible and appropriately represented. It also focuses on the fluidity and naturalness of the actions, ensuring that movements appear realistic and physically plausible, such as GAIA [60] and Human-AGVQA [61]. This aspect is critical for assessing the quality of action representation in human-centric or dynamic videos.

Human artifacts specifically evaluate the representation of human figures, focusing on aspects such as anatomical accuracy, body movement, and gesture. This dimension assesses whether the model accurately generates human figures with correct body proportions and natural movements. It also looks at how well facial expressions and body language are captured, ensuring they align with the actions and emotions intended in the text prompts. In Human-AGVQA [61], this metric is particularly important for videos where human activity is central, ensuring that human figures and their movements are generated authentically.

These special content evaluation dimensions are designed to assess the accuracy and authenticity of content in more complex scenarios, such as videos involving human activities, detailed interactions between objects, or dynamic changes in the scene.

### 3.3. Dataset Descriptions

Recent advancements in text-to-video evaluation have driven the creation of specialized benchmarks addressing distinct aspects of AI-generated video quality. This section systematically analyzes prominent datasets. The comparative of AIGC quality assessment datasets are shown in Table 3.

DEVIL [62] offers 800 prompts across 19 object and 4 scene classes, stratified into five motion levels from static to highly dynamic, to test a model’s ability to manage object motion, action diversity, and scene transitions. AIGV-Assessor [9] contains 36,576 videos from 15 text-to-video models, accompanied by 370,000 expert ratings that cover static fidelity, temporal smoothness, dynamic range, and text alignment through mean-opinion scores and pairwise comparisons. T2VBench [58] provides 1680 temporally rich prompts and 5000 generated videos, enabling evaluation of temporal consistency and complex dynamic rendering via human annotations. EvalCrafter [67] filters 200,000 relevant prompts from 600,000 community submissions, spanning humans, animals, objects, and scenery with style and camera tags, and records user feedback on visual quality, alignment, and motion for model fine tuning. FETV [64] merges 541 MSR-VTT and WebVid text-video pairs with 78 challenging prompts, supporting fine grained spatial and temporal assessment of motion, fluids, and lighting. GAIA [60] comprises 9180 clips from 18 systems covering 510 action classes, and 54 raters score action fidelity, physical plausibility, and continuity with respect to scene context. Human-AGVQA [61] includes 3200 videos generated from 400 prompts, 96,000 quality ratings, and 160,000 semantic labels, focusing on action continuity, body coordination, and scene consistency in human activities. LGVQ [66] offers 2808 clips produced by six leading models from 468 prompts, with ratings for frame clarity, temporal coherence, and text congruence, emphasizing complex motion and scene distortion. MQT [69] supplies 1005 videos from five models driven by 201 prompts and targets semantic alignment and visual realism, especially artifact detection. TC-Bench [65] pairs 150 prompts with 120 image-to-video samples to examine temporal compositionality across attribute shifts, relational changes, and background transitions. T2V-CompBench [59] offers 700 prompts divided into seven categories of 100 each to systematically test multi-object, attribute, and action composition in complex scene generation. T2VQA-DB [68] presents 10,000 videos from nine models based on 1000 prompts, combining large-language-model judgments with classical metrics for the most extensive text-to-video quality evaluation to date.

These datasets provide a wide array of evaluation standards for text-to-video generation, covering everything from basic actions to complex temporal and spatial dynamics. They are invaluable for understanding model performance and guiding improvements in video generation technologies.

## 4. Methodologies for AIGC Video Quality Assessment

### 4.1. Traditional UGC Video Quality Assessment

Video quality assessment has been applied in various fields; most methods [70,71,72,73,74] focus on User-Generated Content (UGC). However, there is still no fair metric for AIGC videos. In previous video generation studies [12,24,27,49], only a few metrics are utilized to evaluate the effectiveness of video generation methods, such as IS [75], FID [76], FVD [77], CLIP [78], CLIPScore [79], and FCS [12]. However, FID, and FVD compare the distribution of Inception [80] features of generated frames with that of a set of real images/videos, thus failing to capture distortion-level and semantic-level quality characteristics. IS [75] does not require reference videos for comparison but relies on pre-trained classification models. Furthermore, motion generation poses a great challenge for current video generation techniques, yet FID [76] and FVD [77] are unable to quantify the impact of temporal-level distortions on visual quality. CLIP-based methods such as CLIPScore [79] and FCS [12] are frequently employed to assess the alignment between the generated video and its prompt text. However, CLIP-based methods can only assess frame-level alignment between the video frames and the text prompt, and they cannot also evaluate the alignment of videos containing diverse motions. It is difficult to rely on these metrics to measure the progress of video generation techniques.

### 4.2. AIGC Video Quality Assessment

The evaluation methods for video quality in AI-generated content (AIGC) across the reviewed texts showcase a variety of advanced techniques, each targeting distinct aspects of video quality assessment, such as spatial, temporal, and semantic alignment, the comparison of AIGC video quality assessment methods in feature and model usage are shown in Table 4.

The AIGV-Assessor, combines 2D and 3D feature extractors, such as InternViT and SlowFast, to capture both spatial and temporal dynamics. The model aligns visual features with textual prompts through MLLM (InternVL2-8B) and follows a multi-stage training process, involving feature alignment, regression fine-tuning, and pairwise comparison optimization. This method evaluates videos across four key dimensions: static quality, time smoothness, dynamics, and text–video alignment.

The MQT [69] employs a dual evaluation framework, using BLIP-2 for text–video alignment and XGBoost to assess the video’s naturalness. Temporal consistency and motion quality are evaluated through pre-trained models for action recognition and optical flow-based analysis. This method aims to improve the accuracy of video assessments by capturing both visual quality and temporal dynamics in generated content.

The VBench [63] framework introduces a comprehensive evaluation that breaks down video quality into 16 dimensions, including object consistency, background consistency, motion smoothness, and dynamic range. It uses feature extraction models like DINO for object consistency and CLIP for background consistency, with temporal analysis based on optical flow. This multi-dimensional approach enables fine-grained analysis of video generation, assessing motion dynamics and video-condition consistency.

DEVIL [62] addresses the unique challenges of evaluating the dynamic quality of AI-generated videos, often overlooked by traditional methods. It defines dynamic range, controllability, and quality based on video dynamics, using multi-granularity dynamic scoring methods such as optical flow, SSIM, and entropy. This method uncovers how AI models often optimize static features at the expense of dynamic consistency, emphasizing the need for more advanced dynamic assessments.

T2VBench [58] introduces a new evaluation framework focusing on temporal dynamics in T2V models. It categorizes video content into event dynamics, visual dynamics, and narrative dynamics, analyzing how well T2V models handle complex time-related transitions. Using models like action recognition and semantic consistency tools, T2VBench evaluates video generation across dimensions such as event sequencing, scene transitions, and emotional changes.

TC-Bench [65] centers its evaluation on the concept of time-composition, assessing models’ ability to generate videos with consistent attributes, object relations, and background transitions. It uses assertion-based evaluation through GPT-4 to verify whether generated videos align with given text prompts, with a focus on complex combinations of actions, such as object interactions and scene transitions.

T2V-CompBench [59] emphasizes the importance of evaluating T2V models’ ability to handle complex dynamic scenes. It utilizes multimodal LLMs for cross-modal matching, combining video tracking with feature extraction to assess attributes, actions, and interactions. This framework exposes the limitations of current models in generating consistent dynamic attributes, such as object motion and interaction, in more complex scenes.

The T2VQA [68] method introduces a large-scale dataset and a multi-modal evaluation model. It assesses videos from two perspectives: text–video alignment and video fidelity, using BLIP for semantic matching and Swin Transformer for evaluating video distortion. Combining both text–video alignment and video fidelity in a unified framework, this method outperforms traditional models, providing a more comprehensive and accurate quality assessment.

FETV [64] addresses fine-grained evaluation by introducing a multi-dimensional classification system. It evaluates T2V models across spatial and temporal content, emphasizing motion dynamics, event sequencing, and prompt complexity. This method introduces improved automatic evaluation metrics like UMTScore and FVD-UMT, offering better alignment with human judgment compared to traditional metrics, delivering more reliable insights into model performance.

These methods, ranging from detailed spatial and temporal assessments to advanced dynamic evaluations, provide a comprehensive and nuanced approach to measuring the quality of AI-generated videos. Each framework introduces innovative techniques to tackle the unique challenges of T2V models, enhancing both the accuracy and reliability of quality assessments.

### 4.3. Technical Trends on MLLMs

#### 4.3.1. Empirical Superiority of MLLM–Based Metrics

**Figure 2:**
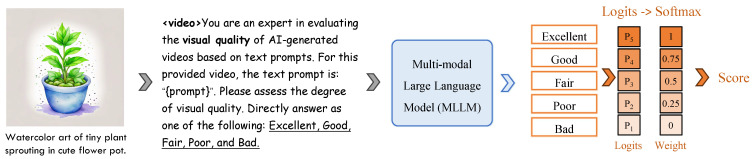
The schematic diagram of the process of visual quality assessment using a multimodal large model inference.

To evaluate the performance of CNN-based, VLM-based, and MLLM-based methods, we fine-tuned each approach on the LGVQ and FETV datasets. The implementation of the MLLM-based method is described below. Our implementation follows a light-tuning strategy that takes advantage of the strong visual priors of a pre-trained MLLM while keeping the computational footprint modest. The flowchart of visual quality assessment using a multimodal large model inference is shown in Figure 2.

- Backbone freezing and partial fine-tuning. The entire vision encoder is *frozen*. We fine-tune only the language model. This choice preserves low-level perceptual features while allowing the linguistic pathway to learn a quality-aware vocabulary.
- Discretised quality tokens. Continuous mean-opinion scores (MOSs) are mapped to five ordinal tokens: <Excellent> (5), <Good> (4), <Fair> (3), <Poor> (2), and <Bad> (1). During training the model is asked, via a masked-language objective, to predict the correct token given the video frames and their generating prompt.
- Score reconstruction at inference. Let $p\;=\;[p_{5},\ldots ,p_{1}]$ be the softmax probabilities over the five tokens. The final quality estimate is the expectation $\hat{q}=5p_{5}+4p_{4}+3p_{3}+2p_{2}+1p_{1}$, i.e., a weighted sum where token weights correspond to their ordinal ranks.
- Uniform frame sampling. Each video is temporally normalized to 32 frames. For clips shorter than 32 frames, we use all available frames; longer clips are uniformly sub-sampled without replacement. Frames are resized to 224×224 and packed into a single visual sequence that the frozen encoder processes in one forward pass.

Table 5 delivers a clear message: once multimodal large language models (MLLMs) are brought into the evaluation loop, they set a new upper bound for every major quality dimension. Relative to the strongest convolutional or vision–language model (VLM) baselines, MLLM metrics achieve uniformly higher rank correlations (SRCC/KRCC) and linear correlations (PLCC) on both LGVQ and FETV. The methods involved in spatial and temporal quality are all the results after fine-tuning, and the methods involved in alignment quality are all zero-shot results.

We adopt a train/validation/test split to retrain all metrics. The final model is selected based on its best performance on the validation set and subsequently evaluated on the test set. The reported results are averaged over 10 trials to ensure a reliable measure of the method’s generalization capability. In our experiments, the data is split into training, validation, and test sets in a ratio of approximately 7:1:2. It is important to note that each dataset contains multiple videos generated from the same prompt. To ensure our method generalizes well to unseen prompts, we adopt an invisible prompt strategy, where no prompt in the validation or test sets appears in the training set. That is, the prompts used for training are completely separated from those used for validation and testing.

- Spatial fidelity. On LGVQ, the leading CNN metric LIQE attains an SRCC of 0.721, whereas Ovis2 and QwenVL2.5 push the score to 0.751 and 0.776, respectively—an absolute gain of 0.03–0.06 (4–8%). A comparable improvement is observed on FETV (0.799 vs. 0.832–0.854).
- Temporal coherence. Motion-aware CNNs such as SimpleVQA plateau at 0.857 SRCC on LGVQ; QwenVL2.5 elevates this to 0.893. On FETV, every MLLM surpasses the best CNN baseline (FastVQA, 0.847), again converging near 0.893.
- Prompt consistency. Alignment is the most challenging axis. Frame-level VLM scores (e.g., CLIPScore) reach only 0.446 SRCC on LGVQ. MLLMs close over one-third of that gap, with DeepSeek-VL2 achieving 0.551; on FETV the margin widens from 0.607 to 0.747, a 23% relative lift.

Across the board, every MLLM driven metric equals or exceeds the best single-modal competitor, and the advantage is most pronounced for semantic alignment: precisely the failure mode that traditional hand-crafted or unimodal features fail to capture. These empirical gains make a compelling case that MLLMs are no longer experimental add-ons but the de facto backbone for next-generation AIGC-VQA. The roadmap below outlines how this transition is likely to unfold.

#### 4.3.2. Roadmap for MLLM-Centric VQA Pipelines

Beyond the current leaderboards, a growing body of work (e.g., LIQE [85], Q-Align [100], AIGV-Assessor [9]) shows that coupling dense vision features with language embeddings enables pixel-to-sentence error localization, natural-language rationales, and rapid domain transfer. We anticipate three converging trends.

1. Multimodal fusion at scale. Future assessors will ingest synchronized visual, textual, and motion tokens, leveraging backbones such as CLIP [101], QwenVL [6], InternVL [7], and GPT-4V [102]. A single forward pass will jointly rate spatial fidelity, temporal smoothness, and prompt faithfulness, eliminating the need for ad hoc score aggregation.
2. Data-efficient specialization. Domain shifts—new genres, unseen distortion types, or language locales—will be handled by lightweight adapters: prompt engineering, LoRA fine-tuning, and semi-supervised self-distillation. These techniques cut annotation cost by orders of magnitude while preserving the zero-shot flexibility of the frozen backbone.
3. Standardization and deployment. Given the quantitative edge illustrated in Table 5, we expect major toolkits to embed an MLLM core within the next research cycle. Stand-alone feature-engineering pipelines will be relegated to legacy status, much like PSNR after the advent of SSIM.

Taken together, the empirical evidence and the emerging tool-chain ecosystem indicate that MLLM-based assessors will dominate the forthcoming landscape of AIGC video-quality evaluation, delivering higher accuracy, richer interpretability, and greater adaptability than conventional CNN or VLM metrics.

#### 4.3.3. Limitations

Despite the promising performance of MLLM-based visual quality assessment methods, several limitations remain. First, MLLMs are prone to hallucinations, responding confidently even when they do not fully understand the input. Although fine-tuning with large-scale instruction datasets and applying Reinforcement Learning from Human Feedback (RLHF) can help mitigate this issue [103], it cannot be entirely eliminated. Second, while these models can generate continuous quality scores by assigning probabilities to quality-related tokens, such score generation may still lack full interpretability or robustness in edge cases. Lastly, over-reliance on MLLM-based systems may lead to rare but extreme failure cases. It should be noted that similar issues are not unique to MLLMs and can also occur in traditional CNN-based quality assessment models.

## 5. Conclusions

AIGC video quality assessment is a complex, multidisciplinary field requiring innovative methods and frameworks to bridge the gap between human perception and technical evaluation. While current models demonstrate substantial progress, challenges in maintaining temporal coherence, action continuity, and dynamic scene accuracy persist. This paper highlights the importance of multi-modal large language models to provide a more holistic and human-aligned assessment of AI-generated video content. Looking forward, we posit that with the continuous advancement of sensors, there will be more modal supporting generative models that are not limited to traditional images, text, audio, video, etc. The generation capabilities of generative models will continue to expand to more modalities, and multimodal large models will further demonstrate their powerful capabilities in the field of multimodal evaluation. 

## Figures and Tables

**Table 1:** Technical specifications of mainstream T2V models.

Model	Time	Duration(s)	Resolution	Frames	Open Source
PixVerse v4 [29]	2025.02	5.0	640 × 640	150	✕
kling v1.6 [30]	2025.01	5.0	1920 × 1080	120	✕
Hunyuan [31]	2024.12	5.4	720 × 1280	129	✓
LTX [32]	2024.11	5.0	1280 × 720	120	✓
mochi-1-preview [33]	2024.11	5.4	854 × 480	162	✓
Haiper [34]	2024.10	5.0	720 × 720	120	✕
CogVideoX1.5-5B [35]	2024.09	5.0	1360 × 768	80	✓
Dreamina [36]	2024.08	5.0	1024 × 1024	120	✕
Gen-3 [37]	2024.07	15.0	1920 × 1080	360	✕
Ying [38]	2024.07	5.0	1280 × 720	120	✕
Latte [39]	2024.05	2.0	512 × 512	16	✓
MagicTime [40]	2024.04	2.1	512 × 512	48	✓
StreamingT2V [41]	2024.03	5.0	1280 × 720	40	✓
open-sora-plan [42]	2024.03	2.7	512 × 512	65	✓
Sora [43]	2024.02	60.0	1920 × 1080	1440	✕
StableVideo [44]	2024.02	4.0	1024 × 576	96	✓
AnimateLCM [45]	2024.02	1.0	512 × 512	16	✓
AnimateDiff [46]	2024.01	2.0	512 × 512	16	✓
VideoCrafter2 [23]	2024.01	1.6	1024 × 576	16	✓
Pika [47]	2023.12	3.0	1280 × 720	72	✕
Show-1 [48]	2023.10	2.4	576 × 320	29	✓
Hotshot-XL [49]	2023.10	2.0	672 × 384	8	✓
LaVie [50]	2023.09	2.4	512 × 320	61	✓
ZeroScope [51]	2023.06	2.0	384 × 384	16	✓
CogVideo [14]	2022.05	2.0	480 × 480	32	✓
Video Fusion [52]	2023.03	2.0	256 × 256	16	✓
Text2Video-Zero [27]	2023.03	3.0	512 × 512	12	✓
text-to-video-synthesis [53]	2023.03	2.0	256 × 256	16	✓
Tune-a-video [12]	2022.12	2.4	512 × 512	24	✓
LVDM [19]	2022.11	2.0	256 × 256	16	✓
NUWA [12]	2021.11	1.0	256 × 256	8	✓

**Table 2:** The comparison of the prompt selection scheme.

Dimension	Synthetic Prompt	Dataset Derivation	Hybrid Method
Source	Artificial and MLLM generation	Existing data set extraction	Artificial and dataset mixing
Focus	Control diversity and reasoning	Real-world relevance	Balance control and authenticity
Advantage	Systematic capability testing	Meets real user needs	Balances diversity and practicality
Disadvantage	Potential intention mismatch	Inherits datasetbias	High complexity

**Table 3:** Comparative analysis of T2V quality assessment datasets.

Dataset	Focus	Videos	Prompts	Models	Annotators
AIGVQA-DB [9]	Perceptual distortions	36,576	1048	15	120
EvalCrafter [67]	Holistic evaluation	5600	700	8	7
FETV [64]	Fine-grained fidelity	2476	619	4	3
GAIA [60]	Action plausibility	9180	400	18	54
Human-AGVQA [61]	Human artifacts	3200	400	8	10
T2VQA-DB [68]	Multi-dimensional	10,000	1000	9	27
T2VBench [58]	Temporal dynamics	5000	1680	3	3
LGVQ [66]	Spatio-temporal	2808	468	6	20
DEVIL [62]	Dynamicity	800	800	3	120
T2V-CompBench [59]	Compositionality	700	700	3	8
TC-Bench [65]	Temporal	150	150	3	3
MQT [69]	Semantic, visual quality	1005	201	4	24

**Table 4:** Comparison of AIGC video quality assessment methods in feature and model usage.

Method	Spatial Quality	Temporal Quality	Text–Video Alignment
AIGV-Assessor [9]	InternViT, SlowFast	InternViT, SlowFast	InternVL2-8B
GHVQ [61]	CLIP-based Action Recognition	SlowFast, Action Recognition	CLIP-based
EvalCrafter [67]	CLIP-based, Action Recognition	Action-Score, Flow-Score	CLIPScore, SD-Score
MQT [69]	BLIP-based, XGBoost	Action Recognition, Optical Flow	BLIP-based
VBench [63]	DINO, CLIP-based	Optical Flow	CLIP-based
DEVIL [62]	-	Dynamic Range, Optical Flow	-
T2VBench [58]	-	Event Dynamics, Action Recognition	Action Recognition, Semantic Consistency
TC-Bench [65]	-	Event Dynamics, Temporal Consistency	GPT-4, CLIP-based
T2V-CompBench [59]	-	Tracking, Action Recognition	GPT-4, MLLM
T2VQA [68]	CLIP-based, Swin Transformer	Motion, Time Dynamics	BLIP-based, CLIP-based
FETV [64]	-	SlowFast, CLIP-based	UMTScore, FVD-UMT
LGVQ [66]	CLIP-based, Vision Transformer	SlowFast, CLIP	CLIP-based, BLIP-based

**Table 5:** The performance of the CNN-based and MLLM-based metrics on the LGVQ and FETV datasets. VLM refers to the vision–language model.

Aspects	Methods	Parameters	Model Type	LGVQ			FETV		
				SRCC	KRCC	PLCC	SRCC	KRCC	PLCC
Spatial	UNIQUE [81]	32M	CNN	0.716	0.525	0.768	0.764	0.637	0.794
	MUSIQ [82]	27M	CNN	0.669	0.491	0.682	0.722	0.613	0.758
	StairIQA [83]	34M	CNN	0.701	0.521	0.737	0.806	0.643	0.812
	CLIP-IQA [84]	0.3B	VLM	0.684	0.502	0.709	0.741	0.619	0.767
	LIQE [85]	88M	VLM	0.721	0.538	0.752	0.765	0.635	0.799
	DeepSeek-VL2 [86]	4.5B	MLLM	0.746	0.549	0.768	0.821	0.664	0.825
	Ovis2 [87]	8B	MLLM	0.751	0.563	0.786	0.834	0.674	0.832
	QwenVL2.5 [88]	7B	MLLM	0.776	0.582	0.802	0.855	0.692	0.854
	InternVL2.5 [7]	8B	MLLM	0.769	0.572	0.797	0.849	0.692	0.845
Temporal	TLVQM [89]	23M	CNN	0.828	0.616	0.832	0.825	0.675	0.837
	RAPIQUE [90]	28M	CNN	0.836	0.641	0.851	0.833	0.691	0.854
	VSFA [91]	31M	CNN	0.841	0.643	0.857	0.839	0.705	0.859
	SimpleVQA [92]	88M	CNN	0.857	0.659	0.867	0.852	0.726	0.862
	FastVQA [93]	18M	CNN	0.849	0.647	0.843	0.842	0.714	0.847
	DOVER [94]	56M	CNN	0.867	0.672	0.878	0.868	0.731	0.881
	DeepSeek-VL2 [86]	4.5B	MLLM	0.868	0.673	0.884	0.868	0.727	0.881
	Ovis2 [87]	8B	MLLM	0.888	0.689	0.903	0.872	0.737	0.886
	QwenVL2.5 [88]	7B	MLLM	0.893	0.694	0.905	0.896	0.751	0.902
	InternVL2.5 [7]	8B	MLLM	0.878	0.683	0.891	0.892	0.739	0.893
Alignment	CLIPScore [79]	0.15B	VLM	0.446	0.301	0.453	0.607	0.498	0.633
	BLIP [95]	0.14B	VLM	0.455	0.319	0.464	0.616	0.505	0.645
	viCLIP [96]	0.43B	VLM	0.479	0.338	0.487	0.628	0.518	0.652
	ImageReward [97]	0.15B	VLM	0.498	0.344	0.499	0.657	0.519	0.687
	PickScore [98]	0.98B	VLM	0.501	0.353	0.515	0.669	0.533	0.708
	HPSv2 [99]	0.15B	VLM	0.504	0.357	0.511	0.686	0.540	0.703
	DeepSeek-VL2 [86]	4.5B	MLLM	0.551	0.393	0.577	0.741	0.573	0.747
	Ovis2 [87]	8B	MLLM	0.554	0.401	0.585	0.750	0.583	0.748
	QwenVL2.5 [88]	7B	MLLM	0.571	0.410	0.592	0.762	0.595	0.764
	InternVL2.5 [7]	8B	MLLM	0.555	0.396	0.583	0.744	0.582	0.745

## Data Availability

Data sharing is not applicable (only appropriate if no new data is generated or the article describes entirely theoretical research).
